# Long-Term Impact of Malaria Chemoprophylaxis on Cognitive Abilities and Educational Attainment: Follow-Up of a Controlled Trial

**DOI:** 10.1371/journal.pctr.0010019

**Published:** 2006-08-18

**Authors:** Matthew C. H Jukes, Margaret Pinder, Elena L Grigorenko, Helen Baños Smith, Gijs Walraven, Elisa Meier Bariau, Robert J Sternberg, Lesley J Drake, Paul Milligan, Yin Bun Cheung, Brian M Greenwood, Donald A. P Bundy

**Affiliations:** 1 Partnership for Child Development, Department for Infectious Disease Epidemiology, Imperial College School of Medicine, London, United Kingdom; 2 Medical Research Council Laboratories, Banjul, Gambia; 3 Center for the Psychology of Abilities, Competencies, and Expertise, Yale University, New Haven, Connecticut, United States of America; 4 Child Study Center, Yale University, New Haven, Connecticut, United States of America; 5 London School of Hygiene and Tropical Medicine, London, United Kingdom; 6 Human Development Network, World Bank, Washington, District of Columbia, United States of America

## Abstract

**Objectives:**

We investigated the long-term impact of early childhood malaria prophylaxis on cognitive and educational outcomes.

**Design:**

This was a household-based cluster-controlled intervention trial.

**Setting:**

The study was conducted in 15 villages situated between 32 km to the east and 22 km to the west of the town of Farafenni, the Gambia, on the north bank of the River Gambia.

**Participants:**

A total of 1,190 children aged 3–59 mo took part in the trial. We traced 579 trial participants (291 in the prophylaxis group and 288 in the placebo group) in 2001, when their median age was 17 y 1 mo (range 14 y 9 mo to 19 y 6 mo).

**Interventions:**

Participants received malaria chemoprophylaxis (dapsone/pyrimethamine) or placebo for between one and three malaria transmission seasons from 1985 to 1987 during the controlled trial. At the end of the trial, prophylaxis was provided for all children under 5 y of age living in the study villages.

**Outcome Measures:**

The outcome measures were cognitive abilities, school enrolment, and educational attainment (highest grade reached at school).

**Results:**

There was no significant overall intervention effect on cognitive abilities, but there was a significant interaction between intervention group and the duration of post-trial prophylaxis (*p* = 0.034), with cognitive ability somewhat higher in the intervention group among children who received no post-trial prophylaxis (treatment effect = 0.2 standard deviations [SD], 95% confidence interval [CI] −0.03 to 0.5) and among children who received less than 1 y of post-trial prophylaxis (treatment effect = 0.4 SD, 95% CI 0.1 to 0.8). The intervention group had higher educational attainment by 0.52 grades (95% CI = −0.041 to 1.089; *p =* 0.069). School enrolment was similar in the two groups.

**Conclusions:**

The results are suggestive of a long-term effect of malaria prophylaxis on cognitive function and educational attainment, but confirmatory studies are needed.

## Editorial Commentary

**Background:** The burden of disease and death from malaria is well documented, but little is known about the impact of malaria on the mental development of children and their ability to learn. Evidence from observational studies suggests episodes of malaria are associated with a negative impact on mental processes such as language, memory, and attention. However, there is very little evidence from trials on whether community-level approaches to malaria prevention can improve mental and educational development in children. In a trial conducted in the Gambia between 1985 and 1987, which was reported in *The Lancet* (21: 1121–1127), young children were allocated either to receive dapsone/pyrimethamine (a commonly used drug for malaria prevention) or placebo, for up to four years. At the end of the trial, the drug was then offered to all children at the study sites. In a follow-up to the original trial, reported here, the researchers then attempted to trace the original trial participants and look at various measures relating to mental development: memory, attention, reasoning, knowledge, language, and level of schooling reached.

**What this trial shows:** The investigators found no significant differences in mental development scores (memory, attention, reasoning, knowledge, and language) between children who had received malaria prevention during the trial and those who had not, although scores appeared higher for children who received malaria prevention for the longest period. However, they did see a significant difference in schooling level, with children who received malaria prevention during the trial having achieved just over half a grade higher in school.

**Strengths and limitations:** The original trial methods ensured that the two participant groups were comparable on relevant demographic, household, and educational factors at the start of the trial. Furthermore, the measures used to compare mental development were appropriate, validated for African populations, and then further adapted in the groups being studied. Although only close to half of the original trial participants could be successfully traced, a sample size calculation shows that the follow-up study probably had enough power to detect important effects. However, a major limitation of the study is that once the main trial was complete, all participants were offered malaria prevention with dapsone/pyrimethamine. This limits the extent to which the follow-up study could have detected prevention effects 14 years later, had they existed. The researchers investigated this by doing separate analyses based on how long children spent in the trial. They found a stronger positive effect of malaria prevention on mental development scores for children who spent longer in the trial (and therefore got post-trial prevention for a shorter period of time). This finding supports, but does not conclusively prove, the hypothesis that malaria prevention enhances mental development.

**Contribution to the evidence:** This study adds data from a well-controlled clinical trial to the body of evidence suggesting that malaria prevention may have beneficial effects on mental and educational development. Very few previous trials have examined this outcome; these results need to be confirmed in future studies specifically designed to test such hypotheses.

## INTRODUCTION

International initiatives to control malaria—such as Roll Back Malaria and the Global Fund to Fight HIV/AIDS, Tuberculosis and Malaria—can be justified in terms of the documented mortality and morbidity due to this disease. Each year African children under the age of 5 y suffer between 400 and 900 million acute febrile episodes [1], and between 700,000 and 2.7 million of them die from malaria [1,2]. In addition, malaria has a considerable social and economic impact [3]. Such analyses, however, may underestimate the burden of malarial disease [4] if they overlook its impact on cognition and education [5,6]. There are few data on which to estimate the burden of cognitive impairment associated with malaria [7].

Evidence suggests that malaria can impair cognitive development [8,9]. Cerebral malaria—malaria accompanied by coma—can cause severe neurological impairment in survivors, including speech and behavioural disorders, hearing impairment, blindness, epilepsy, hemiplegia, and cerebral palsy [10]. Less severe impairments in cognitive functions are also observed in children up to at least 2 y after an episode of cerebral malaria [11–14]. Few studies, however, have addressed the effect of malaria on cognitive abilities at the community level. Two studies have found an association between repeated malaria episodes and poor performance in educational tests [15,16]. Only one study has measured the impact of effective malaria treatment on cognitive function. This study found no overall effect on cognitive function of schoolchildren with asymptomatic Plasmodium falciparum infection 2 wk after antimalarial treatment [17], although treatment appeared to improve visual memory and fine motor control in children with the highest pre-treatment parasitaemia. Overall, such findings are suggestive of an association between malaria and cognitive function. Without supporting data from carefully controlled intervention studies, however, the potential role of confounding factors related to both malaria infection and deficits in cognitive function cannot be excluded. Interventions can also meet the need for community-level data, as opposed to focusing on survivors of cerebral malaria. In addition, there is a lack of evidence on the long-term cognitive effects of malaria, as studies have tracked survivors of cerebral malaria only into the early years of primary school. We aimed to address these gaps by assessing the cognitive abilities and educational attainment of a cohort of young children who had taken part in a malaria chemoprophylaxis trial 14–16 y previously [18].

There is growing evidence that the health and nutrition of young children has a long-term effect on their cognitive development. For example, Giardia lamblia infection in the first 2 y of life is associated with a lower IQ at age 9 y [19]. Children undernourished in the first 2 y of life have lower IQs, lower educational achievement, and higher levels of conduct disorders in adolescence than do well-nourished children [20–23]. Children with iron deficiency in the second year of life are more likely in adolescence to have poor motor, cognitive, and educational outcomes; anxiety and depression; and attentional, social, and behavioural problems [24]. Similarly, malaria infection could have a deleterious effect on cognitive function, as a result of cerebral malaria, anaemia, or malnutrition [9], which might be alleviated by effective malaria control programmes. Thus, we hypothesised that children who had received malaria chemoprophylaxis in early childhood would have improved cognitive abilities and educational attainment in late adolescence.

## METHODS

### Participants

The original malaria chemoprophylaxis trial recruited children aged 3–59 mo of age living in 15 villages situated between 32 km to the east and 22 km to the west of the town of Farafenni, the Gambia, on the north bank of the River Gambia, approximately 100 km from the coast. Malaria and iron deficiency are the main causes of anaemia in children in this area; hookworm is uncommon.

Five of the 15 villages withdrew from the trial before its completion, due to the death or dismissal of the village health worker in two villages and inadequate drug supply in the others. The remaining ten villages were invited to participate in a follow-up study. Participants qualified for the current study if they were eligible to receive placebo or prophylaxis for three consecutive months in at least one of the three final malaria transmission seasons during the original trial. A total of 1,190 participants (604 female) of median age 17 y 5 mo (range 14 y 9 mo to 20 y 3 mo) were eligible for the current study. Of these, 18% (214) had been present in the original trial for 1 y, 39% (464) for 2 y, and 43% (512) for 3 y. The length of time children were present in the original trial depended on their date of birth and when they moved to or from the village.

### Interventions

Children were allocated systematically by residential compound (a group of households) to receive dapsone/pyrimethamine (Maloprim) or a matching, inert placebo. The intervention was given fortnightly during the malaria transmission season to all children present in the village in the eligible age range from April 1984 to March 1988. Compliance was 60% for chemoprophylaxis and 59% for placebo during the last year of the formal surveillance period [25] and a little higher 2 y previously. As a result of reductions in mortality and morbidity attributable to chemoprophylaxis, dapsone/pyrimethamine was offered to all children of an eligible age in study villages at the end of the trial in 1988 through a Ministry of Health primary health-care programme. Chemoprophylaxis was then sustained during the malaria transmission season for at least 2 y in study villages [26], until replaced by insecticide-impregnated bed nets as the primary method of malaria control in the Gambia.

Children from a 1:5 random selection of compounds were evaluated approximately 2 mo before the intervention began and throughout observation periods for the next 4 y. Placebo and intervention groups in this sub-sample were well matched for age and ethnic group. Chemoprophylaxis was highly effective. During two periods of observation [18,25] overall mortality was reduced by approximately 40% and malaria-attributable mortality by about 80% in children who received prophylaxis. In the Farafenni area, children experience on average one clinical attack of malaria every two rainy seasons; this was reduced by about 75% in children who received chemoprophylaxis. The mean packed cell volume was substantially higher in children who had received chemoprophylaxis than in those who had received placebo at the end of both periods of observation (33.9% versus 31.2% for the first period and 33.5% versus 31.9% for the second period), and fewer children who had received chemoprophylaxis were underweight (<80% weight for height) at the end of the rainy season (41% versus 59%).

### Objectives

The objective of the study was to assess the long-term educational and cognitive effects of malaria chemoprophylaxis in early childhood.

### Outcomes

The outcomes of the follow-up study were cognitive function, educational attainment (highest grade of schooling reached), and school enrolment.

Assessment of the impact of chemoprophylaxis on educational performance and cognitive function was not an initial end-point of the trial. However, because of increasing interest in the possible effect of malaria on educational performance and the paucity of information in this area, we considered it worthwhile to trace as many of the children who had participated in this trial as possible and to assess their cognitive ability and past educational performance.

### Sample Size

Power calculations indicated that a sample of 550 participants would be sufficient to detect a 0.3–standard deviation (SD) difference between groups with 80% power, assuming an average of four participants per compound and an intracluster correlation coefficient of 0.2. This effect size is typical of cognitive effects of other early childhood health and nutrition interventions [27].

### Randomisation

Each compound was allocated a three-digit number in a sequential manner moving around the village. Compounds whose number ended in a zero or an even number were allocated to receive placebo, and those whose number ended in an odd number received chemoprophylaxis. Further details of the trial are given in earlier publications [18,25].

### Blinding

All field staff and participants in the original trial and the follow-up study were unaware of the allocation to intervention or placebo group.

### Procedures

As the study villages are included in the Farafenni Demographic Surveillance System [28], participants' records were checked to ensure that their date of birth and parental names matched those in the demographic database maintained at the Medical Research Council.

The follow-up study was approved by the Gambia Government and the Medical Research Council Ethics Committee. Meetings were held in all villages with political leaders and village heads to explain the objectives and methods of the study, to answer questions, and to obtain consent for the study. Written, informed consent was obtained from all participants or from parents or guardians of those under 18 y of age. Data collection took place from May to November 2001.

A battery of cognitive tests was administered to participants. Three tests measured memory and attention. These were a digit span test, assessing short-term memory for strings of orally presented digits (in order of presentation and then, in a separate test, in reverse order), a categorical fluency test, assessing the number of animals and food types children can name in two 1-min sessions, and a visual search test, assessing the speed at which children identify target pictures from amongst distracters. There were two tests corresponding to the two constituent factors of general intelligence—fluid intelligence (inductive reasoning) and crystallised intelligence (knowledge) [29]. Raven's Coloured Progressive Matrices Test assessed children's reasoning ability, and a Gambian adaptation of the Mill Hill Vocabulary Test [30] was a measure of knowledge. There was also a test of proverb understanding based on a sub-test of the Wechsler Adult Intelligence Scale [31] designed to measure verbal ability using culturally relevant stimuli. With the exception of the proverbs test, all cognitive tests had been previously validated with African populations [32], and all tests were adapted for use with the Mandinka and Wollof groups. For tests of verbal ability (the vocabulary and proverbs tests), only questions that were equivalent in the two languages were used. All measures were piloted extensively and tested for validity and test–retest reliability, assessed through correlation between scores from repeated test sessions 1 wk apart. Two tests—the digit span backwards test and the categorical fluency test (food section)—were found to have low reliabilities (<0.65) and were dropped from the test battery. Four cognitive testers were trained and monitored through the use of test–retest reliabilities. Training was complete when all testers achieved acceptable reliability levels (>0.65) in all tests. The test of proverb understanding was further assessed to ensure that coding of responses was consistent across the four testers. Inter-tester reliability, assessed through correlation, was at least 0.96 for all tester pairs.

Tests were administered in one session lasting around 45 min in a quiet area in one compound in each village. All testing was done in the child's language of preference—either Wollof or Mandinka. Children were fed a sandwich before testing to reduce the effects of hunger on performance [33].

### Socioeconomic, Demographic, Anthropometric, and Educational Data

Two field workers administered a questionnaire to all participants to obtain basic demographic information and the educational history of participants and their parents. Height was measured using a portable stadiometer (CMS Weighing Equipment, London, United Kingdom) to a precision of 1 mm. Weight was measured with a mechanical scale. Field workers were trained in anthropometric assessment. In addition, data were available from a previous socioeconomic survey conducted in study villages in 1998. This survey collected data from one representative in each compound, following the methodology of a similar survey [28].

### Statistical Methods

A statistical analysis plan specified the primary end-points of the follow-up study, based on the study protocol. They were cognitive function, school enrolment, and educational attainment (the highest grade of schooling reached). The outcome measure for cognitive function was determined as follows. First, all variables were tested for normality, and one variable (visual search) was Box-Cox transformed to normality [34]. A factor analysis using the maximum likelihood method was performed on the six cognitive function variables: digit span, categorical fluency (animals), visual search, Raven's Matrices, vocabulary, and proverbs. The first factor explained 39% of the variance—the only factor to explain more than one-sixth of the variance. This factor was subsequently used as the sole cognitive function outcome variable in analyses, with higher scores on this variable indicating improved cognitive function. Factor scores were obtained using the Bartlett method [35,36]. The factor loadings of the six variables were fairly similar, ranging from 0.45 to 0.79 (Table S1). The SD of the outcome variable was 1.1.

Regression analysis of cognitive function scores with adjustment for covariates was conducted. The first analysis adjusted for variance due to tester and test language. The second analysis adjusted for the other covariates. Highest education grade was analysed by both linear regression and ordinal logistic regression to see whether the results were model-sensitive [37]. Enrolment in primary school was analysed by logistic regression.

There were no missing cognitive test data for children included in the analyses. There were 93 participants with other missing values in education variables and/or covariates. For analyses that adjusted for covariates, missing values were multiply imputed by iterative multiple regression and the data analysed using multiple imputation methodology [38,39]. On average there were three participants per compound. Huber-White robust standard errors were estimated and used in all inferential statistics to allow for the correlation between participants from the same residential compounds [40].

A secondary analysis examined intervention effects according to the number of years of post-trial prophylaxis received. The length of time eligible for post-trial prophylaxis was a function of children's age at the time of prophylaxis and was independent of children's allocation to trial arms. Eligibility for post-trial prophylaxis was not, however, independent of time on trial—those eligible for the longer periods of post-trial prophylaxis were generally on trial for a shorter period of time (see Results for details). The participants were grouped into four categories according to the number of years for which they were eligible for post-trial prophylaxis: 0 y, 0 to 1 y, 1 to 2 y, and 2 y or more. The regression analyses of cognitive function, highest grade of schooling, and enrolment in formal education were repeated in each of the four categories. The presence of monotonic trends would be taken as evidence of dilution of intervention effects by the availability of post-trial prophylaxis, as indicated by a test of the interaction between intervention group and duration of post-trial prophylaxis in their effects on cognitive function. Finally, interaction between the intervention and gender was explored. All analyses were conducted using Stata version 8 software (StataCorp, College Station, Texas, United States).

## RESULTS

### Participant Flow

**Figure 1:**
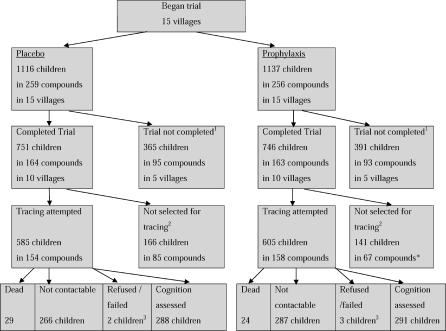
Participant Flow in Follow-Up to Chemoprophylaxis Cluster-Controlled Trial Participant flow in the malaria chemoprophylaxis trial and follow-up 11 y later. Fifteen villages took part in the trial, with treatment allocated systematically by compound. Follow-up was conducted in the ten villages that completed the trial. Children who completed a whole year of the trial were selected to take part in the follow-up. ^1^The village health worker died in one village and was dismissed in another. Drugs were insufficient in three villages. ^2^Only children participating in at least one complete transmission season were selected for tracing. Some compounds contained both children selected and children not selected. ^3^One child refused and one child failed to understand cognitive test instructions in the placebo group. One child refused and two failed to understand instructions in the prophylaxis group. ^*^Intervention group allocation was by compound, trial completion was on a village basis, and selection for tracing was conducted for individual children.

The flow of participants is shown in Figure 1. The proportions of children who were successfully traced and assessed for cognitive function were similar in the prophylaxis and placebo groups (291/605 = 48.1% and 288/585 = 49.2%, respectively; *p =* 0.70).

### Baseline Data

The two intervention groups were also similar in terms of age, gender, ethnicity, duration of both trial participation and eligibility for post-trial prophylaxis, and several economic and anthropometric indicators (Table 1). Only a quarter of fathers and approximately a tenth of mothers had received formal primary education. Children in the chemoprophylaxis arm appeared to be more advantaged in terms of father's education and, to a lesser extent, mother's education. However, they were less advantaged in that fewer of them had a radio in their compounds.

**Table 1:**
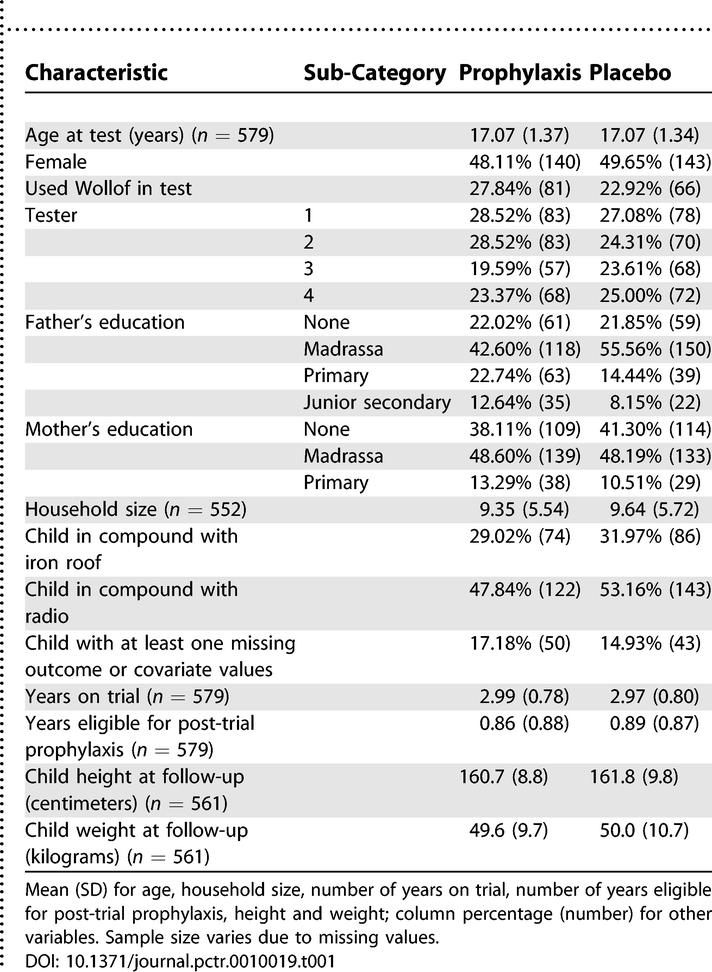
Characteristics of Trial Participants

### Outcomes and Estimation

There were no significant differences in the cognitive function scores between the two arms, with or without adjustment for covariates (adjusted estimates in Table 2; *p* > 0.10 for all; the intracluster correlation coefficient for the cognitive function score was 0.212). The two groups were very similar in the proportion enrolled in school (unadjusted odds ratio = 1.020, 95% confidence interval [CI] 0.658 to 1.583; *p* = 0.928; logistic regression), and adjustment for covariates was without effect (adjusted estimates in Table 2). However, there was a significant (*p =* 0.013) interaction between school enrolment and female gender. In an adjusted model that included this interaction term, the odds ratios (95% CI) on the intervention (control = 0 and prophylaxis = 1), gender (male = 0 and female = 1), and the interaction were 0.674 (0.383 to 1.184), 0.154 (0.087 to 0.272), and 2.773 (1.243 to 6.193). Hence the intervention effect in boys was 0.674 (0.383 to 1.184; *p =* 0.170) and in girls was 0.674 × 2.773 = 1.869 (0.943 to 3.702; *p =* 0.073). The prophylaxis appeared to increase the odds that the girls attended school and decrease the odds that the boys attended school, though these effects did not reach statistical significance.

**Table 2:**
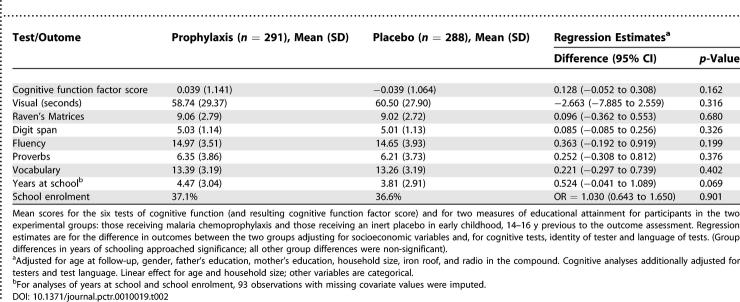
Cognitive Test and Education Outcomes by Intervention Group

The prophylaxis group's mean educational attainment was 4.47 grades at school compared to the placebo group's 3.81. Unadjusted regression analysis gave a mean attainment 0.65 grades higher in the intervention arm (0.023 to 1.276; *p =* 0.042). This estimate was reduced slightly to 0.52 (*p =* 0.069; Table 2) when covariates were included in the analysis. Estimates were similar using ordinal logistic regression (Table S2).

### Ancillary Analyses

Supplementary analyses were conducted, stratified by number of years children were eligible for post-trial prophylaxis. For the cognitive function score, there was a significant interaction between the four sub-groups and the trial intervention (*p =* 0.034), with larger treatment effects for the two groups that spent the longest on trial and received the least post-trial prophylaxis (Table 3). There was a small effect (0.264, or, in units of SD, approximately 0.24 SD, 95% CI −0.03 to 0.51) in the group of individuals that were not eligible for post-trial prophylaxis and spent 3 y in the trial, and a somewhat larger effect (0.43 SD, 95% CI 0.10 to 0.77) among those eligible for less than 1 y of post-trial prophylaxis and who were in the trial for all 4 y. There was no effect among those eligible for more than 1 y of post-trial prophylaxis. For both educational measures, there was no significant interaction between years of post-trial prophylaxis and treatment, but the pattern of results for the highest grade reached (Table 3) was similar to that found for cognitive function, with the largest effects for sub-groups receiving less than 1 y of post-trial prophylaxis.

**Table 3:**
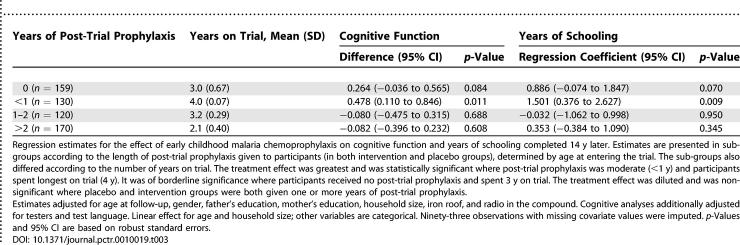
Estimates of Prophylaxis Effect on Cognitive Function and on Years of Schooling, Adjusted for Covariates, by Years of Post-Trial Prophylaxis

## DISCUSSION

### Interpretation

The overall pattern of results is suggestive of a positive long-term effect of the trial prophylaxis on cognitive function diluted by offering prophylaxis to the placebo group at the end of the trial. However, as end-of-trial prophylaxis was not an experimental intervention and was confounded with age and years on trial, it is not possible to draw unequivocal conclusions and confirmatory studies are needed.

There were overall treatment effects of borderline significance on the educational outcome variables. The prophylaxis group had 0.52 more years of schooling (95% CI −0.041 to 1.089; *p =* 0.069). Results suggested that this effect was also diluted by end-of-trial prophylaxis, although the relationship between years of schooling and length of post-trial prophylaxis was non-significant. There was no overall difference in school enrolment; there was a significant interaction between gender and treatment group, but the direction of the effect was different in boys and girls, with girls in the prophylaxis group more likely to enrol in school than girls in the placebo group, and boys less likely. Given international targets of achieving universal primary education by 2015 and gender equity in education by 2005, it is critically important that future studies examine the potential for early childhood malaria protection to contribute to reaching these education goals.

This study has a number of limitations with implications for the interpretation of findings. First, post-trial prophylaxis may have affected results. If the trial intervention had an impact on cognitive and educational outcomes, our results will have underestimated this effect for cohorts who received post-trial prophylaxis. Unfortunately, the effect of post-trial prophylaxis cannot be reliably disentangled from that of the trial intervention. Second, only 50% of target participants were followed up in this study. Follow-up rates and background characteristics were similar between treatment arms in the follow-up sample, suggesting that any treatment effects found were not artefacts resulting from sample bias. Thus, our conclusions stand. However, we cannot be certain that our sample was representative of the original trial population or that our conclusions apply to those children not followed up in this study.

Third, assessing long-term educational outcomes was not an explicit goal of the original trial. Thus, biomedical outcomes were collected only for a proportion of our study sample, and no cognitive assessments were made at the end of the intervention. Such data may have helped clarify the nature of the effect of prophylaxis on cognition.

If treatment effects on cognitive function are genuine, the mechanisms by which chemoprophylaxis achieved these effects can be considered. They are most likely to have arisen from the prevention of cerebral malaria in a small number of children and the prevention of anaemia and improvement of nutritional status in a much larger number. Estimates on the basis of data collected during the period of the trial [18] indicate that prophylaxis led to around ten (~3%) fewer cases of cerebral malaria among the children in our sample, at least 40 (~14%) fewer children with anaemia levels of less than 30% packed cell volume, and more than 50 (~19%) fewer cases of underweight; many more children are likely to have benefited from a more modest increase in their haemoglobin and weight. Malnutrition in early childhood has been shown to have a long-term impact on cognition [19,20,22,23]. The impact of malaria-induced anaemia on cognitive function is poorly understood, but iron deficiency anaemia, which occurs in children with malaria, does have a long-term impact on cognitive function [41,42]. It is also possible that long-term benefits for children's health improved their school attendance, which in turn improved cognitive development [9]. The gender difference in school enrolment, if genuine, could be explained if parents' determination to send girls to school is more vulnerable to setbacks, such as child health problems, than for boys. A similar explanation was put forward for gender differences in the effect of preschool nutrition on school enrolment in Pakistan [43].

### Generalisability

The impact on educational attainment may not be replicated in regions with a higher initial level of primary school enrolment. Conversely, both parasite prevalence and the incidence of clinical malaria are relatively low in the Gambia, and a more marked effect might be observed in an area with a higher level of transmission.

### Overall Evidence

Previous research has found impaired cognitive performance following severe malaria [11–14] and poorer school performance following repeated malaria attacks [15,16]. The current study extends these findings by assessing cognitive function and educational attainment after an experimental intervention; findings are suggestive of a protective effect of malaria chemoprophylaxis on both outcomes. The results further add to existing studies by providing some evidence of cognitive effects of malaria at the population level, rather than just in a sub-population of cerebral malaria survivors, and by finding that such effects persist after 14 y. The study also adds to findings associating malaria with poorer performance in cognitive and educational tests by demonstrating an impact on the highest grade attained at school, an outcome with greater relevance for future life success.

In summary, our study is suggestive of a long-term effect on children's cognitive development and education. Given the prevalence of malaria, any effect it has on cognitive function is likely to amount to a massive cumulative loss across the world. These results draw further attention to the current focus on malaria prevention and treatment in young children, be it by impregnated bed nets, prompt treatment upon infection, or intermittent presumptive treatment for children [44–46]. The study also emphasises the need to investigate the long-term educational and cognitive outcomes of children currently participating in malaria control trials, and helps clarify methodology for such investigations.

The findings also emphasise the potential for health interventions in early childhood to improve cognitive function in the long term. To date, the only early childhood interventions shown to improve long-term cognitive outcomes are those targeted at malnourished young children [20,22,23]. More generally, this study adds to a growing body of research suggesting that one of the most effective ways to improve a child's education is by first improving their health.

## SUPPORTING INFORMATION

CONSORT Checklist Click here for additional data file. (53 KB DOC)

Trial Protocol Click here for additional data file. (57 KB DOC)

Alternative Language Abstract S1 Translation of the Abstract into French by Giorgio Sirugo and Jérôme Feldman (21 KB DOC)

Alternative Language Abstract S2 Translation of the Abstract into Spanish by Conchi Vera-Valderrama and Miguel Vargas-Reus (24 KB DOC)

Table S1 Factor Loadings for the Six Cognitive Tests (27 KB DOC)

Table S2 Estimates of Prophylaxis Effect on Educational Attainment Using Ordinal Logistic Regression (37 KB DOC)

## Abbreviations

CI: confidence interval(s)
SD: standard deviation(s)
