# Supplementary material for: Long-Term Impact of Malaria Chemoprophylaxis on Cognitive Abilities and Educational Attainment: Follow-Up of a Controlled Trial
Source: PLoS Clin Trials. 2006 Aug 18;1(4):e19. doi: 10.1371/journal.pctr.0010019 (PMC1851720; doi:10.1371/journal.pctr.0010019)
Supplement: Trial Protocol [file pctr.0010019.sd002.doc]

Trial Protocol

Medical Research Council Laboratories, Fajara

**Application to undertake a research project**

**_____________________________________________________________________**

#### A Summary Information

**A1 Title of project**

Please choose a short clear title for ease of reference and identification in future.

Long-term cognitive effects of malaria infection in the first few years of life

**A3 Location(s) of research**

Fieldwork will be based in the MRC research station at Farafenni. Data entry will also be in Farafenni. Analysis will conducted in Yale and Oxford Universities.

**A4 Proposed start date and duration in months**

Piloting and preparation will begin in October 2000. The main project will begin in mid-January 2001 and will run for approximately 2 months.

__________________________________________________________________________

**A5 Reference (office use)**

**Scientific Co-ordinating Committee No ....……**

**Ethical Committee No ....……**

**A6 Summary of project, long term objectives and specific aims (not more than 200 words)**

*This section is very helpful to the Committees in determining quickly the main features of the study, and should be as clear and concise as possible. It should cover the key objectives and endpoints and, if the project is hypothesis driven, then the hypothesis should be stated here.*

The aim of the project is to compare psychological outcomes of two groups of children who were given a malaria chemoprophylaxis (Maloprim) or a placebo for up to three consecutive malaria transmission seasons, from 1985-1987 inclusive. A battery of cognitive tests, tests of educational achievement and practical intelligence, and a questionnaire concerning the education history of the child will be administered to all children who took part in the initial randomised trial who can still be contacted.

The hypothesis is that children who received protection from malaria in early childhood will outperform children who received only placebo in all cognitive tests. In particular, tests of educational achievement and other tests measuring abilities influenced by long-term learning will show a greater difference between groups than tests of problem solving and other cognitive tests relatively unaffected by education.

The project will also contribute valuable data on the cognitive and practical abilities of rural African children, and the relationship between these abilities, demographic information and school attendance.

**B Description of Project**

not more than 5 pages covering the following:

**B1 Background**

*The background should show the relationship between the proposed study and the present state of knowledge and should reference previous work by the investigators and others. If you have questionnaires or consent forms prepared, please attach these to the application.*

A number of studies have shown that bouts of cerebral malaria can lead to impaired cognitive function in children 1,2 whilst there is some evidence that less severe, asymptomatic malarial infections are also associated with poorer performance on some cognitive tests 3. Although direct evidence is scant there is good reason to suppose that less severe malaria infection affects cognitive function. Other parasitic diseases, such as helminthiasis, have been implicated as the cause of impaired cognition 4. And two potential mediators of this effect - undernutrition and anaemia - have both been shown to impair cognitive function 4 and can both result from infection with malaria 5,6. Thus, potential pathways exist for uncomplicated malaria to impair cognition.

If malaria does affect mental function, it is likely to have its greatest effect during infancy as the brain is developing. Both IDA and undernutrition have a large impact on cognitive development during this period 7,8. Evidence suggests that interventions taking place during infancy and the preschool years can have very long-term consequences. Iron supplementation during infancy is correlated with improved performance six years later 9 whilst the positive impact of preschool education can be seen more than 20 years later 10. In addition, cognitive performance in infancy is a relatively good predictors of intellectual abilities later in life 11.

Taken together, this evidence suggests that malaria infection during the first few years of life may have significant and long-term effects on the cognitive abilities of children. This has serious implications for control programs. Given the extent of malaria and the likelihood of repeated reinfection with the disease, any effect it has on cognitive function is likely to amount to a massive loss of intellectual potential across the world.

##### B2 Project description

*This should cover project plan, time-scales, description of methods, justification, analyses to be carried out, outcomes (see also B3 where particular details need to be set out and cross refer as necessary).*

The long-term cognitive effects of malaria infection occurring in the first few years of life can be investigated by studying children who took part in a chemoprophylaxis study in the Farafenni region of The Gambia 12. Children between the ages of 6 months and 5-and-a-half years were randomly selected to receive either Maloprim (a malaria prophylactic consisting of pyrimethamine and dapsone) or a placebo once a week for the 20-week duration of the rainy season in 1985. They received similar treatments during the rainy seasons of the two following years. Chemprophylaxis reduced clinical malaria by 74% and increased mean haematocrit by 2.7% 5,13. These children are still under demographic surveillance and in December 1996, MRC staff were able to identify 698 children who took part in this chemoprophylaxis study for 3 consecutive malaria transmission seasons and who were still living in one of the study villages.

It is our hypothesis that children who received anti-malarial drugs in the initial trial will obtain higher scores in various psychometric tests than those who received a placebo. In particular, tests of achievement (such as education tests, vocabulary tests) which are the result of intellectual application over a number of years, are expected to show the greatest differences between groups. We also predict smaller differences in tests of other cognitive abilities.

This project also builds on existing work 14 looking at the interrelation between the abilities of the child, the demographics of the family and school attendance. This work has included the development of novel tests of practical intelligence in children. The current project will extend this work to a new population (all work so far has been conducted in East Africa) and will also assess the impact different methods of schooling (Koranic school vs. Primary School) on cognitive abilities.

**Issues concerning the selection of tests**

*Capturing a range of abilities*

On the basis of current evidence it is difficult to predict which cognitive abilities are likely to be affected by malaria. Thus it is important to test for as broad a range of abilities as possible. The selection of the tests for this study is driven by two prominent theories of cognitive abilities and intelligence. First, the information processing account of human cognition 15 supposes a number of functionally distinct units within the brain which deal with different tasks. Some of the tests in the current study have been selected to measure performance in all the key cognitive domains in this theory. Short-term memory is measured by the digit span test, speed of processing is measured by the picture search task and speed of access to long-term memory is assessed by the verbal fluency test.

The second theory, the triarchic theory of intelligence 16 has also influenced the selection of tests. This theory proposes three relatively independent aspects to human intelligence. Problem-solving ability (termed *fluid intelligence*) concerns the ability to reason and is relatively unaffected by a person’s state of knowledge. This will be measured using Raven’s coloured progressive matrices tests. *Crystallised intelligence* refers to abilities that depend on a person’s state of knowledge, how much they have learnt. This will be assessed by a vocabulary test and a test of arithmetical ability. The final part of the triad is *practical intelligence* which refers to the ability to adapt to one’s environment. This will be assessed using a test developed and piloted specifically for this study.

*Cultural validity*

There are two issues here. First, tests should measure the same abilities in rural Gambians as they do in other cultures. All of the tests (with the exception of the practical intelligence test) were developed in Western settings and it is possible, for example, that a test that measures short-term memory in British children may measure ability to use a pencil, or the degree to which children are comfortable in a testing situation, with a rural Gambian population. To avoid some of these problems, all tests are given orally, in the child’s first language and great care is taken to make the child feel as comfortable as possible before the test begins. All tests used in this study have been used in rural African settings before and can be demonstrated to have a good validity in a number of cultures.

The second issue concerns the relevance of the abilities which are to be tested. A test may have a high validity (in that it measures the intended ability), but may test an ability which is not valued by the local populations. This issue is more difficult to address but is also less problematic. It could be argued that if malaria impairs short-term memory (for example), this is a cause for concern regardless of the importance placed on this ability by the local community.

*Effects of Education*

Participants in this study vary greatly in the amount of education they have received. 31% have attended primary school with 63% having attending Koranic school. Therefore, it is important, both for the validity of the testing procedure, and in order to reduce variance, that tests are included in the battery which do not depend greatly on education. The Raven’s matrices test, in particular, is designed to be free of the effects of education and culture, and the coloured version of this test is less dependent than other forms on the verbal ability of participants. All other tests have also been used successfully with African children who have never attended school 17.

It is also important to include tests which do depend on education levels. One of the hypotheses of the current study is that some of the cognitive abilities affected by malaria may be those that are important for children’s learning. Thus we might expect the gap between the two groups (those affected and unaffected by malaria in infancy) to widen as the learning process continues (whether through formal education or as part of a more general accumulation of experience). According to this line of argument, those tests which depend on learning (and thus on level of education) are most likely to be related to malaria infection in infancy. In this study, the vocabulary and arithmetical ability tests are those which depend most on learning.

*Reliability*

It is important that tests have a high level of reliability. That is, when the same test is administered repeatedly to one child, whether by the same or different testers, the results from each round of testing correlate highly with each other. All tests selected for this study have been shown to have a high reliability when administered to rural African children.

**Procedure**

Testing will take place in a central building in each village. Children will be collected from their homes by a field assistant who will prepare children for tests, give them something to eat and administer two questionnaires: An assessment of Practical Intelligence (the ability to adapt to their environment) and a questionnaire about their education history (number of years attended, level reached). They will also measure the height and weight of the child. In addition, data is available on the socio-economic status of all children in the study. We would expect the two groups to have similar levels of socio-economic status assuming the randomisation process in the initial clinical trial was successful, and subsequent drop-out (presumably due to migration) has not biased the sample in any way. School attendance should be considered an outcome variable as well as a mediator for other cognitive measures. That is, poor school attendance may result from cognitive impairments (or other health-related consequences) resulting from infection and it may also contribute to cognitive impairments.

After answering questionnaires children will have a short break before completing a battery of psychometric tests, administered by one of four cognitive testers (two working in each village at any one time) in a single one-hour session. The battery will consist of a number of tests all of which have been shown to be appropriate for use in a range different countries (including many in Africa) and which do not normally require extensive training of testers. The tests we will use are: the Raven Coloured Progressive Matrices test (a test of fluid intelligence), a locally constructed adaptation of the Mill Hill Vocabulary test (a proxy for crystallised intelligence), an arithmetic test, a measure of digit span (short-term memory), verbal fluency (speed of access to semantic memory) and a speed of processing task.

**Piloting**

All tests would need to be piloted to assess their suitability for use with the local population. Villages included in the study are populated by three ethnic groups - Mandinka, Wolof and Fula - and all tests should be conducted in the child’s mother tongue. The vocabulary test will be constructed in the child’s first language. Tests of arithmetic and digit span should be adapted for base 5 counting systems (as in both Wolof and Fula languages).

**Analysis**

Analysis will be a one-way Analysis of Covariance, with Treatment (Maloprim vs. Placebo) as the single between-subjects factor and each cognitive test as the outcome measure. In addition, school attendance will be used as an outcome measure. In the first analyses, age, sex and SES will be covariates. The additional inclusion of school attendance as a covariate will help determine whether protection from malaria affects cognition beyond any mediating effect of reduced school attendance.

**Timetable**

This is a provisional timetable

October 1^st^ 2000 - Piloting, recruitment and test development.

November 27^th^ 2000 - Obtaining consent during Ramadan (when testing is not possible).

January 1st 2001 - 8 weeks of testing begins

March 1st 2001 - Provisional date for end of testing

**References.**

1. Dugbartey AT, Spellacy FJ, Dugbartey MT. Somatosensory discrimination deficits following pediatric cerebral Malaria. *American Journal of Tropical Medicine and Hygiene* 1998;**59**(3)**:**393-396.

2. Holding PA, Stevenson J, Peshu N, Marsh K. Cognitive sequelae of severe malaria with impaired consciousness. *Trans R Soc Trop Med Hyg* 1999;**93**(5)**:**529-34.

3. Sororis. The effect of asymptomatic malaria on cognitive function: University College London, 1999.

4. Drake LJ, Jukes MCH, Sternberg RJ, Bundy DAP. Geohelminthiasis (ascariasis, trichuriasis and hookworm): Cognitive and developmental impact. *Pediatric Infectious Disease* in press.

5. Greenwood BM, Greenwood AM, Bradley AK, et al. Comparison of two strategies for control of malaria within a primary health care programme in the Gambia. *Lancet* 1988;**1**(8595)**:**1121-7.

6. D'Alessandro U, Olaleye BO, McGuire W, et al. A comparison of the efficacy of insecticide-treated and untreated bed nets in preventing malaria in Gambian children. *Trans R Soc Trop Med Hyg* 1995;**89**(6)**:**596-8.

7. Palti H, Pevsner B, Adler B. Does anemia in infancy affect achievement on developmental and intelligence tests? *Hum Biol* 1983;**55**(1)**:**183-94.

8. Grantham-McGregor S, Walker S, Chang S. Nutritional deficiencies and later behavioural development. *Proceedings of the Nutrition Society* 2000;**59:**1-8.

9. Lozoff B, Jimenez E, Wolf AW. Long-term developmental outcome of infants with iron deficiency [see comments]. *N Engl J Med* 1991;**325**(10)**:**687-94.

10. Karoly LA, Greenwood P, W, Everinghma SS, et al. Investing in our children: What we know and what we don't know about the costs and benefits of early childhood interventions. Santa Monica, CA: RAND, 1998.

11. McCall RB, Carriger MS. A meta-analysis of infant habituation and recognition memory performance as predictors of later IQ. 1993.

12. Allen SJ, Snow RW, Menon A, Greenwood BM. Compliance with malaria chemoprophylaxis over a five-year period among children in a rural area of The Gambia. *J Trop Med Hyg* 1990;**93**(5)**:**313-22.

13. Menon A, Snow RW, Byass P, Greenwood BM, Hayes RJ, N'Jie AB. Sustained protection against mortality and morbidity from malaria in rural Gambian children by chemoprophylaxis given by village health workers. *Trans R Soc Trop Med Hyg* 1990;**84**(6)**:**768-72.

14. PCD. The relationship between child's ability and parental attitude to school attendance in Zanzibar. In preparation.

15. Baddeley A. Working memory. 1986.

16. Sternberg RJ. Beyond IQ: A triarchic theory of human intelligence. 1985.

17. PCD. How schooling affects cognitive function in rural Tanzanian Children. In preparation.

18. Pollitt E. Iron deficiency and cognitive function. *Annu Rev Nutr* 1993;**13:**521-37.
