# Supplementary material for: Long-Term Impact of Malaria Chemoprophylaxis on Cognitive Abilities and Educational Attainment: Follow-Up of a Controlled Trial
Source: PLoS Clin Trials. 2006 Aug 18;1(4):e19. doi: 10.1371/journal.pctr.0010019 (PMC1851720; doi:10.1371/journal.pctr.0010019)
Supplement: Alternative Language Abstract S1 — (21 KB DOC) [file pctr.0010019.sd003.doc]

**Impact à long terme de la chimiprophylaxie anti-paludéenne sur les capacités cognitives et niveau d'éducation aquis: suivie d'un essai contrôlé**

**Objectif:** Évaluer l’impact à long terme du paludisme sur les capacités cognitives de jeunes enfants et le niveau d’éducation acquis.

**Méthode:** Essai contrôlé randomisé basé au niveau des ménage-foyers.

**Sites:** 15 villages repartis sur la rive nord du fleuve Gambie (32km a l’est et 22km a l’ouest de la ville de Farafenni).

**Participants:** 1190 enfants âgés de 3-59 mois ont participé à l’essai. 579 participants ont été retrouvés (291 et 288 repartis dans les groupes chimioprophylaxis et placebo, respectivement); l’âge médian des participants était de 17 ans et 1 mois (âges compris  entre 14 ans 9 mois et 19 ans 6 mois).

**Intervention:** Les participants ont reçu de la MaloprimR (chimioprophylaxie) ou du placebo pendant trois saisons de transmission paludéenne,  de 1985 à 1987. A la fin de l’étude, la chimioprophylaxie a été mise à la disposition de tous les enfants de moins de 5 ans résidant dans les villages.

**Mesures d’efficacité:** Les capacités cognitives**,** l’inscription scolaire, et le niveau d’éducation acquis.

**Résultats** : Aucun effet significatif global de l’intervention sur  les capacités cognitives  n’a été détecté ; en revanche nous avons montre une interaction significative entre le groupe intervention et la durée de la chimioprophylaxie post intervention  (p<0.05). Ces capacités cognitivés étaient plus élevées chez  les enfants  n’ayant pas reçus de la chimioprophylaxis après l’étude (effet de traitement =0.2SD, 95%CI -0.03 SD   to 0.5 SD  ) ainsi que chez les enfants ayant reçu de la chimioprophylaxie pendant moins d’un an après l’étude, comparés aux enfants du groupe placebo. En moyenne, le groupe intervention avait un niveau scolaire 0,52  fois plus élevé (95% CI=-0.041 to 1.089; *p=*.069).  L’inscription scolaire était la même dans les deux groupes.

**Conclusion** : Les résultas suggèrent un effet  significatif  a long terme de la chimioprophylaxie antipaludéenne sur les  capacités cognitives et  sur  le niveau d’éducation acquis,  mais des  études  supplémentaires sont  nécessaires pour confirmer ou réfuter ces résultats.
