# Supplementary material for: Long-Term Impact of Malaria Chemoprophylaxis on Cognitive Abilities and Educational Attainment: Follow-Up of a Controlled Trial
Source: PLoS Clin Trials. 2006 Aug 18;1(4):e19. doi: 10.1371/journal.pctr.0010019 (PMC1851720; doi:10.1371/journal.pctr.0010019)
Supplement: Alternative Language Abstract S2 — (24 KB DOC) [file pctr.0010019.sd004.doc]

**El impacto a largo plazo de quimioprofilaxis para la malaria en las habilidades cognitivas y el rendimiento educativo. Seguimiento de un ensayo de intervención**

**Resumen**

**Objetivos:** Investigar el impacto a largo plazo de la profilaxis contra la malaria en la ninez sobre las capacidades cognitivas y educativas.

**Diseño:** Ensayo de intervención controlado por clúster llevado a cabo en los hogares de los sujetos de experimentacion.

**Lugar donde se realizo la prueba:** 15 pueblos situados entre 32 Km. al Este y 22 Km. al Oeste de la ciudad de Farafenni, al norte de la ribera del río Gambia.

**Participantes:** 1.190 niños de edades comprendidas entre 3-59 meses tomaron parte en el ensayo. De ellos, 579 (269 del grupo de profilácticos y 288 del grupo de placebo) fueron monitorizados en el 2001. En ese momento, la media de edad era de 17 años y un mes (rango 14 años y 9 meses – 19 años y 6 meses).

**Metodo/prueba:** Los participantes recibieron quimioprofilacticos contra la malaria (Maloprim) o placebos entre una y tres temporadas de transmisión de malaria entre 1985-1987 como parte de un ensayo controlado. Al final del ensayo, se distribuyeron profilácticos a todos los niños menores de 5 años de edad que vivían en los pueblos incluidos en este estudio.

**Resultados a medir:** Habilidades cognitivas, inscripción escolar y logro educativo (la nota mas alta alcanzada en la escuela).

**Resultados:** El ensayo no mostro un efecto significativo en las habilidades cognitivas, pero hubo una interacción significativa entre el grupo de intervención y la duración del tratamiento quimioprofilactico tras el ensayo (*p*=0.034), con una habilidad cognitiva de alguna manera mas alta en el grupo de intervención entre los niños que no recibieron ningún profiláctico después del ensayo (efecto del tratamiento = 0.2 desviación estándar (DS), 95% CI -0.03SD to 0.5DS) y entre los niños que recibieron menos de un año de tratamiento profiláctico después del ensayo (efecto del ensayo =0.4SD, 95% CI 0.1 a 0.8). Se observo un mayor rendimiento escolar en el grupo de intervención de 0.52 puntos ((95% CI= -0.041 a 1.089; *p=*.069). La escolarizacion fue similar en los dos grupos.

**Conclusión:** Los resultados sugieren que hay un efecto de los profilácticos contra la malaria sobre la función cognitiva y en el rendimiento educativo pero estudios adicionales son necesarios para poder confirmarlo.
