# Supplementary material for: Long-Term Impact of Malaria Chemoprophylaxis on Cognitive Abilities and Educational Attainment: Follow-Up of a Controlled Trial
Source: PLoS Clin Trials. 2006 Aug 18;1(4):e19. doi: 10.1371/journal.pctr.0010019 (PMC1851720; doi:10.1371/journal.pctr.0010019)
Supplement: Table S1 — (27 KB DOC) [file pctr.0010019.st001.doc]

Table S1. Factor loadings of the six cognitive tests on the single cognition factor

| Cognitive Test | Factor Loading |
| --- | --- |
| Digit Span | 0.51 |
| Verbal Fluency | 0.45 |
| Visual Search | 0.71 |
| Raven Matrices | 0.60 |
| Vocabulary | 0.79 |
| Proverbs | 0.60 |
