# Supplementary material for: Long-Term Impact of Malaria Chemoprophylaxis on Cognitive Abilities and Educational Attainment: Follow-Up of a Controlled Trial
Source: PLoS Clin Trials. 2006 Aug 18;1(4):e19. doi: 10.1371/journal.pctr.0010019 (PMC1851720; doi:10.1371/journal.pctr.0010019)
Supplement: Table S2 — (37 KB DOC) [file pctr.0010019.st002.doc]

The long term impact of malaria chemoprophylaxis

on cognitive abilities and educational attainment:

follow-up of a cluster-controlled trial – supplementary information

Table S2. Estimates of prophylaxis effect on highest grade of education using ordinal logistic regression models, with and without adjustment for covariates.

| Year of post-trial prophylaxis | Data | Ordinal logistic regression | | |
| --- | --- | --- | --- | --- |
|  |  | Odds ratio | (95% CI) | P-value |
| None | Unadjusted | 1.448 | (0.809 to 2.591) | 0.174 |
|  | Adjusted* | 1.659 | (0.922 to 2.986) | 0.091 |
| Less than 1 | Unadjusted | 2.563 | (1.230 to 5.053) | 0.007 |
|  | Adjusted* | 2.396 | (1.096 to 5.244) | 0.029 |
| 1 to 2 | Unadjusted | 1.009 | (0.524 to 1.943) | 0.979 |
|  | Adjusted* | 0.958 | (0.494 to 1.859) | 0.899 |
| 2 or more | Unadjusted | 1.298 | (0.753 to 2.239) | 0.348 |
|  | Adjusted* | 1.294 | (0.751 to 2.232) | 0.354 |

* Adjusted for age at follow-up, gender, father’s education, mother’s education, household size, compound with iron roof and compound with radio. Linear effect for age and household size; other variables are categorical.

P-values and 95% CI based on robust standard errors.
